# Evaluation of Effective Class-Balancing Techniques for CNN-Based Assessment of Aphanomyces Root Rot Resistance in Pea (*Pisum sativum* L.)

**DOI:** 10.3390/s22197237

**Published:** 2022-09-24

**Authors:** L. G. Divyanth, Afef Marzougui, Maria Jose González-Bernal, Rebecca J. McGee, Diego Rubiales, Sindhuja Sankaran

**Affiliations:** 1 Department of Biological Systems Engineering, Washington State University, Pullman, WA 99164, USA; 2 Department of Agricultural and Food Engineering, Indian Institute of Technology Kharagpur, Kharagpur 721302, India; 3 The Institute for Sustainable Agriculture, Spanish National Research Council, 14001 Cordova, Spain; 4 Grain Legume Genetics and Physiology Research Unit, US Department of Agriculture-Agricultural Research Service (USDA-ARS), Pullman, WA 99164, USA

**Keywords:** plant breeding, disease identification, deep learning, generative adversarial networks

## Abstract

Aphanomyces root rot (ARR) is a devastating disease that affects the production of pea. The plants are prone to infection at any growth stage, and there are no chemical or cultural controls. Thus, the development of resistant pea cultivars is important. Phenomics technologies to support the selection of resistant cultivars through phenotyping can be valuable. One such approach is to couple imaging technologies with deep learning algorithms that are considered efficient for the assessment of disease resistance across a large number of plant genotypes. In this study, the resistance to ARR was evaluated through a CNN-based assessment of pea root images. The proposed model, DeepARRNet, was designed to classify the pea root images into three classes based on ARR severity scores, namely, resistant, intermediate, and susceptible classes. The dataset consisted of 1581 pea root images with a skewed distribution. Hence, three effective data-balancing techniques were identified to solve the prevalent problem of unbalanced datasets. Random oversampling with image transformations, generative adversarial network (GAN)-based image synthesis, and loss function with class-weighted ratio were implemented during the training process. The result indicated that the classification F1-score was 0.92 ± 0.03 when GAN-synthesized images were added, 0.91 ± 0.04 for random resampling, and 0.88 ± 0.05 when class-weighted loss function was implemented, which was higher than when an unbalanced dataset without these techniques were used (0.83 ± 0.03). The systematic approaches evaluated in this study can be applied to other image-based phenotyping datasets, which can aid the development of deep-learning models with improved performance.

## 1. Introduction

Aphanomyces root rot (ARR), caused by the oomycete *Aphanomyces euteiches* Drechs. in pea (*Pisum sativum* L.), results in severe root damage, thus reducing pulse quality and yield [1]. Plants are susceptible to this disease during any stage of their growth and development. Seed treatments and fungicides are not completely effective, and the pathogen can survive in the soil for many years without a host. Once the pathogen builds up in the soil due to favorable conditions, it can cause damage to successive susceptible crops as well [2]. Initially, the lateral roots are prone to the infection, and eventually spread to the epicotyl. The pathogen can spread up to a distance of 18 cm from the infected plant and affect nearby healthy plants [3]. The disease may cause loss of crop up to 86% [4]. Thus, the development of resistant cultivars is crucial to limit yield losses.

Breeding and phenotyping have assisted in developing cultivars with better resistance to diseases [5,6,7,8]. Often, the assessment of disease resistance traits (phenotypes) for a broad set of genotypes is performed by observing their visual features [9,10]. However, since large numbers of plant materials are evaluated during cultivar development, standard phenotyping methods can be tedious and sometimes subjective. As an alternative approach, these visual characteristics can be processed for quantitative selection of disease resistance through deep learning-based image processing techniques such as convolutional neural networks (CNNs) [11,12]. Phenotypic features such as the disease status, morphology, and growth dynamics can be extracted automatically by assimilating prior knowledge and expertise [13].

Deep learning has demonstrated its potential in numerous applications of machine vision—classification, object detection, semantic segmentation, and regression tasks [14,15]. Numerous CNN-based deep learning models have been developed for classification purposes. A typical CNN is designed usually using the following: a convolution layer, which extracts features from the input or previous layers; a pooling layer, which generalizes the features and minimizes the size for computational performance; and a fully connected layer, which classifies an image. The convolutional layers [16] are defined by the convolution filters, which help in transforming and highlighting the patterns in the input image. The pooling layers reduce the dimensions of the data by linking a cluster of neurons from the previous layer to a single neuron. The image classification then takes place in the fully connected layers, where the activations are processed in the form of flattened matrices.

Deep learning models have gained popularity in dealing with agricultural problems such as crop and weed species identification [17], plant disease detection [18], fruit counting and grading [19], food and grain quality monitoring [20], yield prediction [21], and crop stress phenotyping [22,23]. Phenomics techniques integrated with deep learning approaches can increase the throughput of plant phenotyping. Transforming the acquired images into authentic, reliable, and wide range of phenotypic features is a key factor for the successful application of image-based tools. Numerous approaches based on CNNs have been proposed by researchers for performing image-based plant phenotyping. An open-source tool called the Deep Plant Phenomics was introduced to implement CNNs for performing several common phenotyping tasks [24]. An accuracy of 96.88% was obtained for classification of five different mutants of Arabidopsis, and a mean absolute difference of 20.8 h was observed for age regression task (prediction of crop age, measured in hours after germination to relate it to plant maturity). A deep learning technique was used to identify the plant stress level due to nitrogen deficiency, in which the CNN outperformed machine learning algorithms and had an accuracy of approximately 75% [25]. A digital plant phenotyping platform for early-stage drought detection and quantification in Arabidopsis was designed using deep learning and chemometrics [26]. The researchers processed close range spectral images with deep learning techniques and validated its feasibility based on an experiment for drought stress quantification in semi-controlled environments.

In this study, a CNN based classification model, DeepARRNet, was implemented to facilitate the evaluation of resistance to ARR in pea cultivars. Visible symptoms of ARR include honey-brown discoloration of pea roots, poor lateral root growth with minimal root hairs, and wilting of lower leaves [1]. The reliability of identification of diseases in crops and severity prediction have improved with the application of deep learning algorithms. However, acquisition of massive amounts of data is a laborious and skill-demanding task [27]. In addition, in many situations, image data for phenotyping are often not balanced between classes, where fewer images may be available in some classes. This situation is sometimes referred to as imbalanced or unbalanced data in data analytics. In existing plant phenotyping studies that are based on deep learning approach, the model does not reflect the features of the minority class owing to an under-sampling problem. Therefore, a proper data balancing technique should be utilized to develop a robust model that can replicate the original form of the unbalanced image data.

The random resampling method has been extensively applied in other fields such as toxicology [28], biotechnology [29], and drug discovery [30] to deal with unbalanced data. In a study on tomato disease detection [31], a deep learning model was used in conjunction with generative adversarial networks (GANs) [32] for generating synthetic images of tomato plants to increase the amount of image data. The model was able to achieve a 10-class classification accuracy of 97.1% and concluded that augmentation through GANs increases the generalizability of the model and prevents it from over-fitting problem. On a similar note, Giuffrida et al. [33] and Espejo-Garcia et al. [34] proposed GAN models to synthesize artificial images of Arabidopsis plants and tomato plants for augmentation purposes. In the former study, the GAN was conditioned by leaf count, generating a plant image with the specified number of leaves. The feasibility and benefits of GAN-based image augmentation for multiple-disease identification were also assessed [35]. The deep learning model achieved an accuracy of 93.7% when trained with both real and GAN-synthesized images. Madsen et al. [36] also applied GAN to generate images of multiple plant species seedlings using a single network for improving the performance of plant species classification models and found better results with an average recognition accuracy of 58.9% for the generated images. Nevertheless, the benefit of the GAN approach over other resampling approaches needs to be further evaluated prior to its application. Therefore, in this study, three class-balancing techniques were enforced to identify the effective technique for improving the DeepARRNet model performance to evaluate ARR disease severity in peas. The three techniques used to address class asymmetry were: (i) random oversampling with image geometry and intensity-based transformations, (ii) synthesizing artificial images for class with low sample size using GAN, and (iii) loss function with class weighted ratio.

The main contributions of the presented work are listed as follows: (i) agriculture data is often limited by small and unbalanced sample size, and the validation of different approaches and its effect on the results is critical information that may be useful to those in the agricultural domain; (ii) the applications of machine learning and/or deep leaning approaches in root sample analysis are highly sparse, though several can be found for crop and leaf samples; and (iii) disease resistance is an important trait that plant breeders need to measure, given that root phenotyping for disease resistance is still based on visual estimation, image-based approaches such as one developed in this project (RGB imaging with CNN-based approach) can be useful.

## 2. Materials and Methods

### 2.1. Sample Preparation and Data Collection

In greenhouse conditions, 50 advanced breeding lines, two cultivars and two John Innes accessions of peas (*Pisum sativum* L.) were evaluated for reaction to a pure culture isolate of *Aphanomyces euteiches*, Dresch. acquired from the USDA-ARS Grain Legume Genetics and Physiology Research Unit, Pullman, WA, United States. The greenhouse was maintained at 25 °C (day) and 18 °C (night) with a 16-h day. Two treatments, control and inoculated, were used and the experiment was planted in a split-plot design (treatment was the whole plot) with three replicates. Zoospores preparation procedure is reported in Wicker et al. [37]. The inoculum concertation was 1 × 10^4^ spores per mL. The major steps involved: (i) disinfection of seeds and planting in containers with perlite as the growing media; (ii) inoculation (2 mL of inoculum to produce infection and 2 mL sterile distilled water for non-inoculated control) performed in fourteen-day-old seedlings; and (iii) evaluation of disease symptoms on cleaned roots by scoring on a 0–5 disease scale, a standard phenotyping procedure reported in McGee et al. [6]. Table 1 describes the symptoms for the visual scores. More details can be found in Marzougui et al. [38].

A digital camera with 16-MB (Canon^®^ PowerShot SX530 HS, Irving, TX, United States) was used to collect image data of 4608 × 3456 pixels at 50 cm above the samples. A fluorescent light source was used to illuminate the object of interest (400–700 nm), and the set-up was similar to those described in Marzougui et al. [38,39]. The original data captured images of six plants together in a single shot with an image resolution of 0.17 mm/pixel. Image acquisition of roots and visual scoring were performed immediately after plants were removed from the pots and roots were cleaned. The images were cropped such that each image comprised of one root sample.

**Figure 1:**
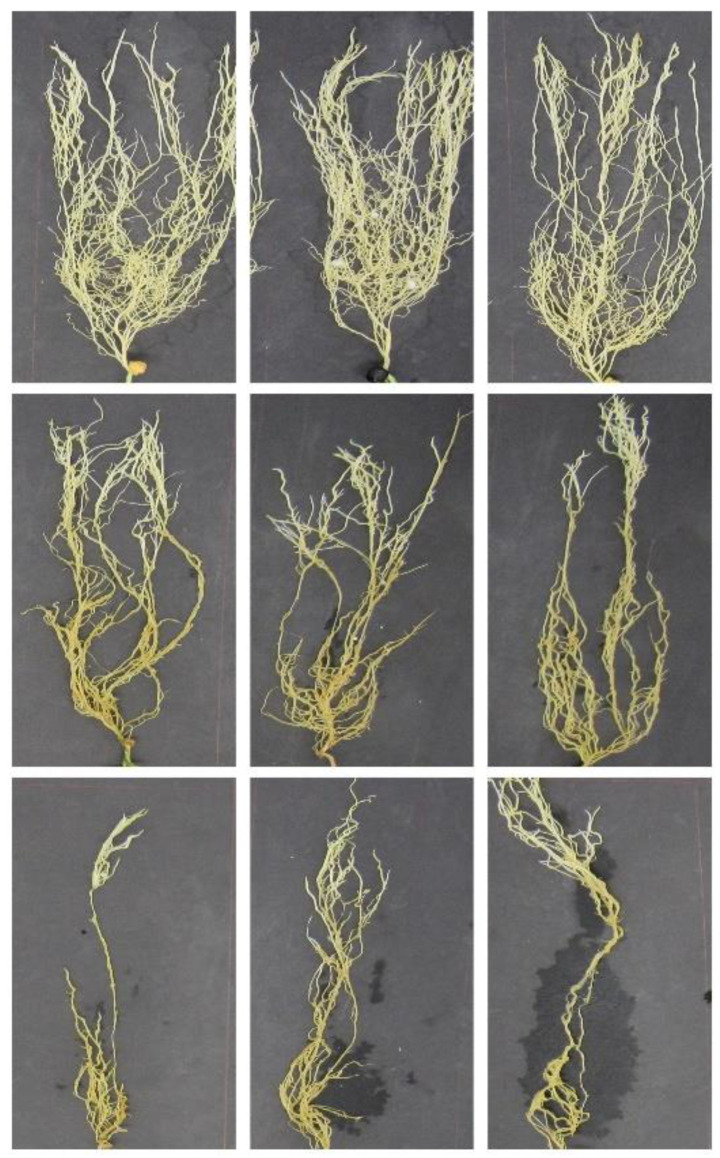
Sample pea root images from the three classes: healthy/resistant (**first row**), intermediate (**second row**), and susceptible (**third row**).

The disease symptoms were rated on a scale from 0.0 to 5.0 through visual inspection of root discoloration and hypocotyl softness. Most of the healthy roots were scored as 0.0, however, the class contained a few root images with a score of 0.5. The disease samples were separated into three classes based on the visual scores: resistant (term generally refers to high levels of partial resistance), intermediate (term generally refers to low levels of partial resistance), and susceptible classes. Since the resistant class had only 4 samples, the final data (1581 non-inoculated and inoculated root images) considered for this study were categorized as resistant (784 images, since the symptoms would be similar to those of non-inoculated root images), intermediate (727 images), and susceptible (70 images) classes. Sample pea root images from the three classes are presented in Figure 1.

### 2.2. Dataset Pre-Processing and Class Balancing

All image processing and analysis were performed in MATLAB^®^ (2021a, The MathWorks, Natick, MA, USA). The program was operated on an Acer Nitro 5 Intel Core i5 9th Generation Laptop (Santa Clara, CA, USA; 32 GB/1 TB HDD/Windows 10 Home/GTX 1650 Graphics). The images were resized to 224 pixels × 224 pixels × 3 bands to fit the input size of the DeepARRNet classification model. The number of images in the resistant and intermediate classes was greater than the susceptible class. Such unbalanced classes may create issues since the model might not learn sufficient features of the specific class of interest (i.e., susceptible). This potential issue, the ‘accuracy paradox’, leads to a better overall performance, even if the result for the susceptible class is poor. Additionally, after the separation of test data, the amount of training data left in these classes is reduced, making it extremely difficult to build a robust model. Therefore, to address this problem, three different class-balancing methods were adopted in this study: (i) increasing the number of images in the underlying class (susceptible) through random oversampling with conventional intensity- and geometry-based image augmentations; (ii) artificially creating additional training images for the susceptible class through GANs; and (iii) modifying the standard loss function of CNN with the introduction of class-weight ratio. The original dataset and datasets created by corresponding methods described above are denoted as S_1_, S_2_, S_3_, and S_4_ hereafter. Each dataset was separately used to train and test the classification models: without class-balancing (using S_1_), and with the above three balancing techniques (using S_2_, S_3_, and S_4_, respectively). Twenty percent of the images in each of these datasets (S_i_; i = 1 to 4) were reserved for testing (T_i_; i = 1 to 4) and the remaining were used for training and developing the model (R_i_; i = 1 to 4).

#### 2.2.1. Random Oversampling

In random oversampling, the images in the underlying class are randomly selected, duplicated, and added to the class’s training data. Since the dataset in this study is highly unbalanced, images of the susceptible class were chosen randomly with replacement, i.e., the same image can be chosen more than once for duplication. However, seeking a balanced distribution by such a resampling operation for highly skewed distribution can result in overfitting problems and reduced generalizability [28]. Hence, instead of adding the duplicated images directly into the training data, image intensity- or geometry-based transformations were additionally performed on these images. These transformations included mirroring along *y*-axis (vertical flipping), translation (left and right) along *x*-axis by a specified number of pixels, Gaussian blurring with a standard deviation of 1.5, and brightness variation with propositional coefficients of 0.85, 0.95, and 1.15. Therefore, for each image from the training set S_2_ considered for oversampling, seven augmented images were additionally derived. Finally, to reduce the class imbalance in the dataset, 600 images for the susceptible class were derived by this resampling method and added to the training set of S_2_ (R_2_) to support the model in training process.

#### 2.2.2. GAN-Based Image Augmentation

The GAN architecture consists of a generator for synthesizing new images, and a discriminator that differentiates these synthetic images from the real ones [32]. The features of the output image are conditioned by the real images used for training the model. The generator and the discriminator undergo simultaneous training in an adversarial process, where the generator tries to deceive the discriminator through its artificial images, while the discriminator diagnoses these artificial images.

The main goal of developing a GAN was to generate artificial pea root images similar to the real images with ARR infection based on the specific class. The resulting images were used to augment the S_3_ training set (R_3_). Thus, the role of the artificially generated images was to increase the number of training samples in the underlying class, i.e., susceptible class, which was expected to improve the classification accuracy of the model.

The proposed generator network accepts random 100-dimensional vector *z* and upscale into an array with the size of 24 × 24 × 512 using a fully connected operation in the first step. This array is passed through a set of four transposed convolutional (t-Conv) layers, with each of the first three followed by a batch-normalization layer and a ReLU layer. The t-Conv layers use 5 × 5 filters and 2 × 2 strides to perform transposed convolutions. For the last t-Conv layer, three 5 × 5 filters were specified, which corresponds to the three channels in the RGB images. The network outputs pseudo root images *G*(*z*) the size of 224 × 224 × 3, with similar visual features to that of the original images. The input to the discriminator network is the generated *G*(*z*) and the original images *x*. This network optimizes its parameters and weights to improve its ability to correctly identify the input image as real or artificial. The ultimate goal of the generator is to produce a data distribution *G*(*z*) very close to *x*, expressed mathematically by the logarithmic function *log*(1 − *D*(*G*(*z*))), where *D*(*G*(*z*)) is the discriminator’s output. Thus, a smaller value of this function denotes better performance of the generator. On the other hand, the optimization goal of the discriminator is to precisely determine if its input is from *G*(*z*) or *x*, given by *log*(*D*(*x*)).

**Figure 2:**
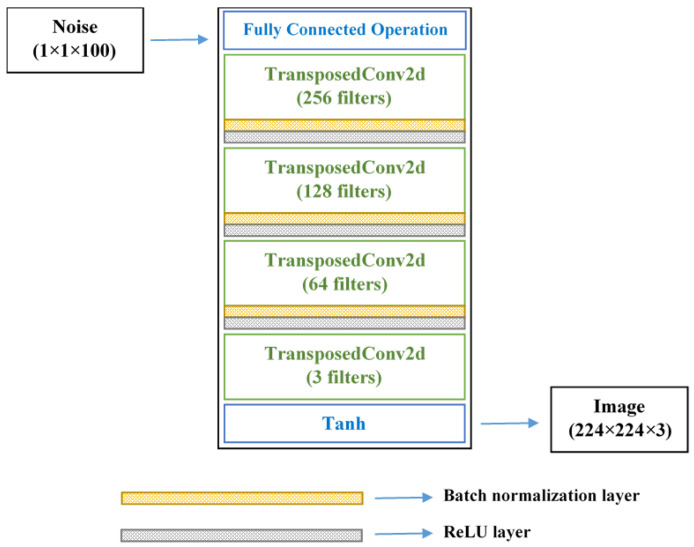
Network architecture of GAN-generator model.

**Figure 3:**
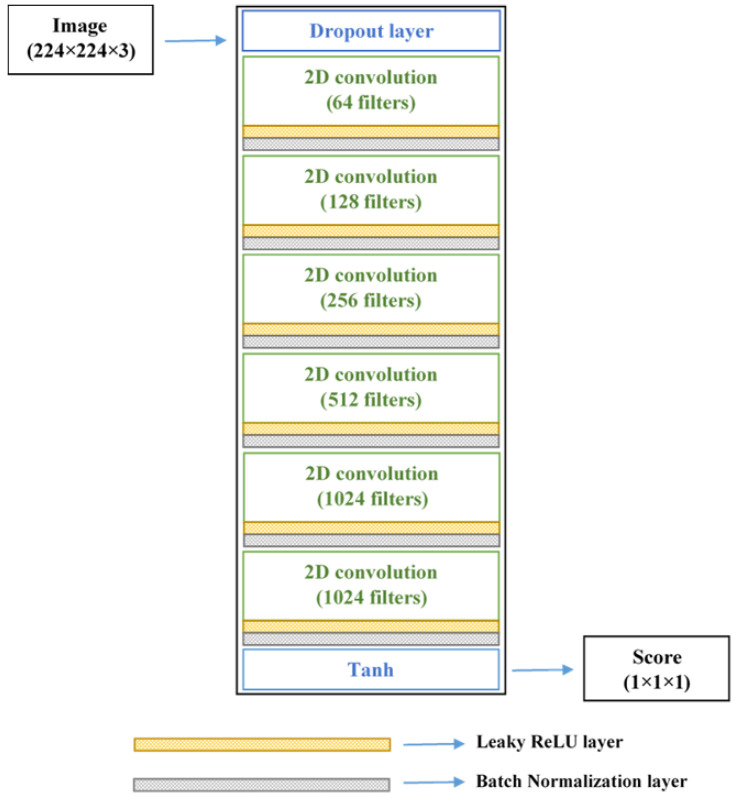
Network architecture of GAN-discriminator model.

The discriminator returns a prediction score (whether the image is recognized as real or synthetic) using a series of convolution, batch normalization, and leaky ReLU layers. Convolution parameters specified for the discriminator were similar to the generator’s t-Conv layers: 5 × 5 filters and 2 × 2 strides. In addition, the discriminator was fitted with leaky ReLU (with a scale of 0.15) in place of ReLU, and a dropout layer (probability of 0.3) to add noise to the input image. The use of batch-normalization layers stabilizes the network, preventing it from crashing during the training process. The tanh function was used at the last layer of the generator and discriminator networks. The detailed architectures have been illustrated in Figure 2 and Figure 3, respectively (Tables S1 and S2 in Supplementary Materials provide the summary of the networks). After some iterations, the loss function scores of the generator and discriminator will reach an equilibrium, after which the generator can be expected to synthesize plausible images from random vectors.

The equations defining the objective function of GAN, where the discriminator tries to maximize this function against the adversarial generator that tries to minimize it, can be found in Madsen et al. [36]. The Adam optimizer with a learn rate of 0.001 and gradient decay factor of 0.5 was set as the optimization algorithm to update the weights of the GAN. The training was manually stopped after 800 iterations. The original images of the susceptible class were fed to the GAN model and a total of 600 artificial images were generated, which combined, made the training set (R_3_).

#### 2.2.3. Loss Function with Weighted Ratio

The loss function is a primary key for training any deep learning model with high performance and robustness. In this study, we implemented a commonly used loss function for classification problems—the multi-class cross entropy loss function which combines the multi-class cross entropy loss with the sigmoid activation layer. Since the frequency of appearance for susceptible class during training was much less compared to the other two classes (resistant and intermediate), using the standard loss function makes the classification model tend to learn the features only from the dominant classes, ignoring the underlying susceptible class. As a modification, the loss computed for the samples was weighted based on the number of samples in each class. Intuitively, higher weight was assigned to the loss experienced due to the misclassification of samples in the minor class. For a given batch size *N,* number of samples *n* in a batch, and class number *c* (*c* = 1, 2 or 3), the weight assigned for the class, *w_(n,c)_* is given by equation described in [22]. Two weighing schemes were used to compute the sample weights: (i) inverse of number of samples (INS); and (ii) inverse of square root of number of samples (ISRNS), described in Equations (1) and (2), respectively.

(1) $$w_{n,c_{INS}}=\frac{1}{Number\;of\;samples\;in\;class\;c}$$

(2) $$w_{n,c_{ISRNS}}=\frac{1}{\sqrt{Number\;of\;samples\;in\;class\;c}}$$

The dataset S_4_ was used to evaluate the classification performance with these weights incorporated in the neural network’s loss function.

### 2.3. DeepARRNet Architecture

During our preliminary evaluation, various state-of-the-art CNNs such as VGG16, Resnet51, Inceptionv3, Xception, and EffiencientNet-B0 were evaluated, where EffiencientNet-B0 outperformed other models. Therefore, in this study, the proposed DeepARRNet network was developed based on EfficientNet-B0 [40] classification model. The researchers observed that better accuracy can be achieved by stabilizing the network’s depth, width, and resolution. Increasing the depth can help the network learn complex features and increase generalization ability; wider networks can learn finer details in the image; and in a high-resolution image, the minute details are plausible. Hence, harmonizing the scaling of these three dimensions of a CNN is important to achieve improved accuracy. Based on this observation, the EfficientNet family of networks has been developed to improve the performance by adopting a fixed set of scaling coefficients for scaling in all three dimensions—depth *α* (number of channels), width *β* (number of layers), and resolution *γ* (number of pixels in the image). A compound coefficient *ϕ* was defined that denotes the quantity of resources available to determine the scaling of *α*, *β*, and *γ*. The restraint (*α* × *β*^2^ × *γ*^2^) ≈ 2 is enforced to make sure that the total floating-point operations per second (FLOPS) does not exceed 2*ϕ*. In the DeepARRNet model, the parameter values are *α* = 1.1; *β* = 1.2; and *γ* = 1.15. The accuracy and FLOPS are together optimized through this multi-objective based neural architectural search.

**Figure 4:**
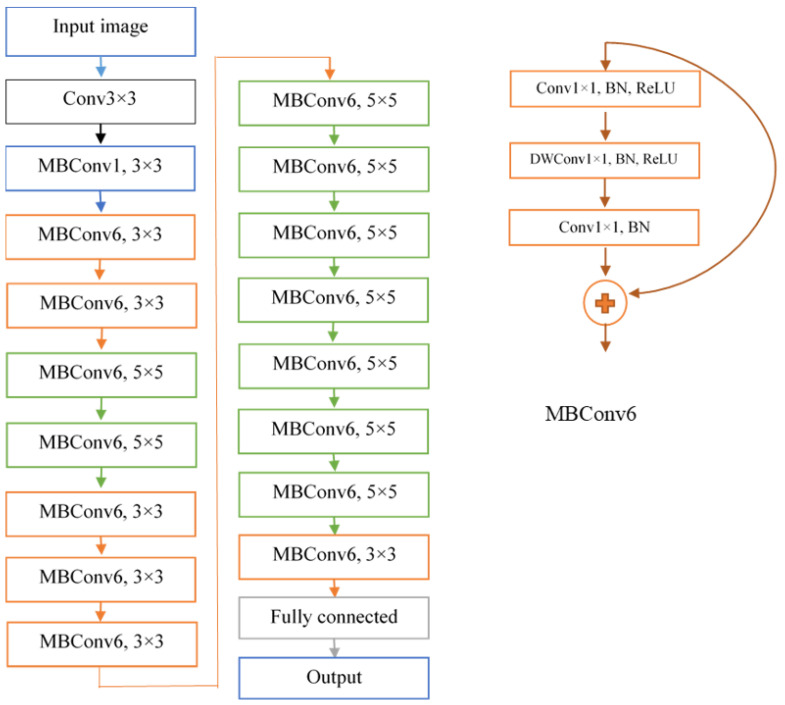
Schematic representation of DeepARRNet.

The network comprises ‘inverted’ residual blocks, sometimes called MBConv (Mobile Inverted Bottleneck Convolution), which was introduced in the MobileNetv2 CNN architecture. The residual block concatenates the activations in the start and end of a convolutional block through a skip connection. The initial layer with more channels is compressed using 1 × 1 convolution operation, and then expanded at the end to match with the number of channels in the initial layer (for concatenation), whereas in inverted residual blocks, the network is widened in the first step by 1 × 1 convolutions, followed by a depth-wise convolution, and in the final step, another 1 × 1 convolution reduces the network to fit the original number of channels. As mentioned earlier, all images were resized to a dimension of 224 × 224 pixels to fit the input size of the network. The overall structure of the proposed model, which classifies pea root images into either ‘intermediate’, ‘susceptible’ to ARR infection, or ‘resistant’, is presented in Figure 4. The Softmax function was used as the activation function at the last layer of the model. For the DeepARRNet model trained with different methods of balancing the data, the stochastic gradient descent with momentum (sgdm) was adopted as the optimizer for training the networks, with a mini-batch size of 16 images and maximum number of epochs set to 30 (with early stopping). Other hyperparameters were optimized separately with each of the four datasets (R_1_—without class balancing and standard loss function; R_2_—classes balanced through oversampling; R_3_—classes balanced through GAN-synthesized images, and R_4_—unbalanced classes with weighted loss function) using the trial-and-error method on the following set of values—learn rate: (0.001, 0.005, 0.01, 0.05, 0.1, 0.5); momentum: (0.9, 0.99, 0.999); and learn rate drop factor for a period of 20 iterations: (0.001, 0.005, 0.01, 0.05).

The DeepARRNet model was evaluated under four conditions based on different class-balancing techniques. After tuning the hyperparameters for each of the four conditions independently, the network was trained and tested for three independent runs (to avoid the effect of single random sampling on the model performance) on the corresponding dataset (with different seeds). The procedure is summarized in Table 2. The precision, recall, accuracy, and F1-score evaluation metrics were used to statistically analyze the performances. Precision is the ratio of true positives and total number of classified objects, while recall is the ratio of true positives and the actual number of samples in the evaluated data set. The F1-score is defined as the harmonic mean of precision and recall. Accuracy is the percentage of samples correctly classified by the model. The testing results are reported in the paper, whereas the training results are summarized in the Supplementary Materials (Tables S3–S6).

## 3. Results

### 3.1. Performance of the Model Using Original Images

The DeepARRNet was initially evaluated with the original dataset to determine the potential of the model for classifying disease resistance to ARR. After tuning the hyperparameters, this model was trained and tested independently on S_1_ with three seeds. The class-wise and overall classification results (Mean ± SD) on the test data are presented in Table 3. It was observed that the model that was trained and validated with the original data had an overall F1-score of 0.83 and an average accuracy of 84.4%. For this model, the class-wise F1-score was 0.95 for classifying resistant root images and 0.88 for intermediate. The precision values ranged from 0.80 to 0.99, whereas recall ranged from 0.06 to 0.99. As expected, though good results were obtained for the resistant and intermediate classes, the performance for classifying images in the underlying susceptible class was low, with an F1-score of only 0.09. This potential problem (accuracy paradox) resulted in a good overall performance but poor results over the classes with a smaller number of samples. Balancing the classes with an efficient method could accord a robust classification model, hence, can improve the performance of the DeepARRNet model during classification.

**Figure 5:**
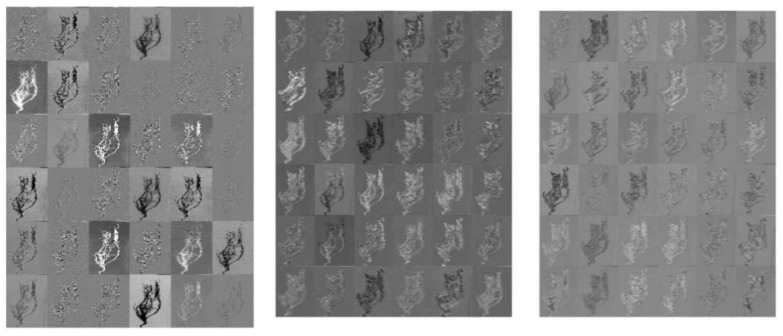
Activation maps of the input image from the DeepARRNet model.

The activation maps derived from the intermediate layers of the network are illustrated in Figure 5. The maps present the first 36 features from the first, the penultimate, and the last convolution layer of DeepARRNet (from left to right in Figure 5). It can be observed that the model tends to learn finer and minute details present in the images as the layers get deeper. The activations just present the outlines of the roots in the initial layers, whereas, in deeper layers, the feature maps seem to be more abstract and have no sharp edges. The activations gradually fade, which means that the disease portions of the root get more attention rather than just the edges.

Skewed distribution of images over the classes (such as the dataset used in this study) are very commonly encountered in plant phenotyping studies. These results imply that models trained with unbalanced datasets are not suitable for screening plant cultivars based on the severity of disease. Since the performance of the model was not satisfying over a particular class when trained with such a dataset, this study also investigated the impact of three data-balancing methods on the model’s performance.

### 3.2. Impact of Random Oversampling Method on Model Performance

The disproportionateness in the pea root dataset was reduced by oversampling the images in the susceptible class randomly with additional image augmentations, thereby creating 600 new images for the class to support the training. The model performance on the test sets is reported in Table 4. It can be observed that the recall value for susceptible class has improved to 0.68 from just 0.06 (without balancing), thus improving the F1-score of the class. For the model, the F1-score was 0.78–0.96, precision was 0.86–0.99, and recall rate was 0.68–0.98. The overall F1-score and average classification accuracy were 0.91 and 91.9%, respectively. The results showed that random resampling (oversampling) method with added image geometry and intensity-based augmentations significantly improved the overall results of the model.

### 3.3. Impact of Addition of GAN-Generated Images on Model Performance

**Figure 6:**
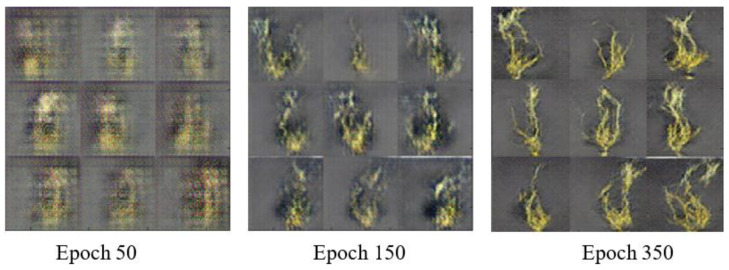
Synthetic ARR-affected pea root images generated by GAN model during the training process.

**Figure 7:**
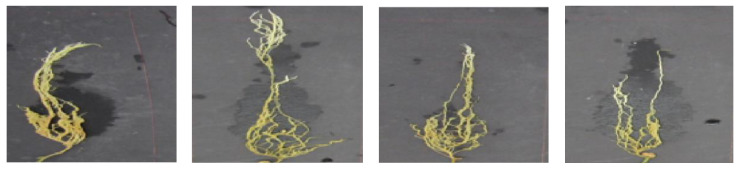
Sample artificial images synthesized by the GAN-generator model.

The GAN model proposed was also implemented for generating artificial images of the susceptible class using the available original images in the training set (R_3_). The fidelity of the generated images was assessed through visual analysis before adding them to train the DeepARRNet model. Figure 6 presents the artificial images produced at different epochs of GAN’s training process. The training was manually stopped after 800 epochs (~5500 iterations). From the training plot (associated with the generator and discriminator scores), it was observed that an equilibrium was reached soon after 3000 iterations. This shows that feasible numbers of features have been learnt by the network and can now generate plausible images of pea roots affected by ARR. After completion of the training phase, the generator component of the GAN was used to create new artificial images of the susceptible class by passing random vectors. The qualitative results of the generator can be visually examined in Figure 7.

The classification performance of DeepARRNet after training with the unified original and GAN-generated images are presented in Table 5. It is evident from the table that data augmentation through GANs to balance the dataset can cause a performance boost for the underlying class as well as the overall results. After training on the combined dataset, the F1-score and accuracy of the model improved progressively to 0.92 and 93.3%, respectively. Moreover, the recall rate and F1-score of the susceptible class was observed to be 0.75 and 0.81, respectively. This demonstrates that the GAN-generated images create emphasis and provide more information on the features of this class, making the model more robust.

In this study, the GAN was able to generate representative images for susceptible class after training it with 70 original images (Figure 6 and Figure 7). This could be because of the dataset being collected in a controlled environment, where imaging conditions were optimized and stable. Therefore, there were lower variations in image characteristics due to illumination, background, the orientation of the root (aligned along *y*-axis), etc. Hence, the features representing the class were learnable during GAN application with a lower number of images. Nevertheless, more training data might improve the quality of the generated images, associated features, and results.

### 3.4. Impact of Introducing Class-Weighted Ratio in Loss Function

In this section, two class weight ratio schemes namely, INS and ISRNS, were investigated on the loss function to determine the best approach for dealing with unbalanced classes. The metrics in the test datasets are shown in Table 6. The overall F1-scores in both cases exceeded 0.87. It shows that class weights can increase the performance of the model as the recall rates were observed to be around 0.60, compared to the 0.06 when no class balancing technique was adopted. Furthermore, weighing the loss function as an INS method gave a slightly better result as compared to ISRNS. For the DeepARRNet model implemented using INS weighing scheme, the F1-score was 0.78–0.96, precision was 0.88–0.99, and recall rate was 0.64–0.98. Interestingly, the precision value of classifying susceptible pea root images (0.89) was similar to the intermediate class (0.88). This shows that the class weighting method influences the model such that the model learns from the features of all classes with equal priority.

## 4. Discussion

Deep learning algorithms can facilitate quantifying disease resistance in crops, as in this study, where DeepARRNet was used to evaluate the ARR resistance in pea cultivars. The model was developed to provide an end-to-end assistance to classify pea roots among three ARR severity classes: resistant, intermediate, and susceptible. An overall F1-score of 0.83 was observed, although the susceptible class accuracies were low. This can be anticipated due to the unbalanced distribution of image data, especially in the underlying class, though the overall performance was acceptable.

Unbalanced classes are a common issue for the application of deep learning algorithms, especially in the agricultural domain [41,42,43]. One of the major objectives in this research was to evaluate multiple class balancing approaches to mitigate the problem with unbalanced class sizes, especially since there may be some overlap between the intermediate and susceptible classes on visual characteristics. All the three approaches utilized in this study (random oversampling-based image augmentation, GAN based image augmentation, and inclusion of weighing functions during classification) improved the overall performance of DeepARRNet. Amongst these results, the GAN-based image synthesis of a susceptible class showcased a highest overall F1-score of 0.92. The GAN-based approach may be computationally intensive, depending on data size, image resolution, and GAN network. The benefits of GAN-based image synthesis in improving model performance should surpass its limitations for successful implementation. Thus, it should be noted that the significance of selecting the effective class balancing technique would depend on the characteristics of the dataset, deep learning model, and the optimization techniques adopted. Previously, Marzougui et al. [39] adopted a CNN-based model and machine learning algorithms of selected image features to evaluate the severity of ARR infection in lentils. The generalized linear regression model resulted in an accuracy of up to 91% for classification of three disease severity classes. Many studies in the literature have dealt with similar problems using hyperspectral imagery. For instance, Nagasubramanian et al. [44] deployed a novel CNN model that had a classification accuracy of 95.7% to identify the soil borne fungal disease charcoal rot in soybean crops using hyperspectral images.

Rebalancing the dataset can change the decision boundaries of the classification model, thus improving the classification accuracies. This increases the chances of resulting in a better performance by converting the false negatives into appropriate predictions [29]. This will improve the recall rate of the underlying class, as observed in this study (comparing the results in Table 3 with the performances when class-balancing was implemented, i.e., in Table 4, Table 5 and Table 6). Zhou et al. [45] reported that combining GAN with classification network improved the average recall rate by 19% for identifying five stored-grain insect species. Similarly, there was a significant improvement in accuracy (+5.2%) when GAN-generated images were used to support the training of tomato disease identification model [46]. However, there is a risk of decrease in precision value due to misclassification of negative samples as false positives. This theoretical intuition was in par with the results of this experiment, as the precision value of susceptible class decreased when the dataset balancing was attempted. Thus, class balancing improves the decision boundary, associating positive and negative samples into positive note. This slightly reduces the precision but can boost the recall rate, hence improving the F1 score.

## 5. Conclusions

Deep learning-based techniques show encouraging results in the agricultural domain. This study proposed a CNN-based deep learning model—DeepARRNet—for qualitative analysis of resistance to ARR in pea cultivars. The pea root image dataset comprising three classes (“resistant”, “intermediate”, and “susceptible”) corresponding to the severity of infection was prepared to train the proposed model. Since the dataset was highly unbalanced, three class balancing techniques were compared based on the classification performance of the model. The F1-scores obtained with the original unbalanced dataset, through random oversampling, GAN-based image synthesis, and with class-weight ratio implemented in the loss function were 0.83, 0.91, 0.92, and 0.88 respectively. All three approaches were successful in improving the F1 score of the weakest class (susceptible class had least samples) from 0.09 in unbalanced dataset to about 0.78–0.81. Therefore, the study highlights the need for a suitable data-balancing techniques to develop a robust prediction deep learning model for agricultural and phenomic applications. In future, diverse datasets (different growing conditions, multiple image resolutions, and other imaging conditions) may need to be utilized to further validate the applicability of the evaluated approaches.

## Figures and Tables

**Table 1:** Aphanomyces root rot visual disease scoring criteria.

Visual Disease Score	Symptoms	Class	Number of Image Samples
0.0	No discolored lesions on the entire root	Healthy/Resistant	784
0.5	Up to 5% of discolored lesions on the entire root	Resistant	4
1.0	5–15% of discolored lesions on the entire root		
1.5	15–25% of discolored lesions on the entire root		
2.0	25–50% minor discoloration on the entire root	Intermediate	727
2.5	50–75% major discoloration on the entire root		
3.0	More than 75% of brown discoloration on the entire root		
3.5	More than 75% of brown discoloration on entire root system with some symptoms on hypocotyl	Susceptible	70
4.0	Brown discoloration on entire root system with shriveled and brown hypocotyl		
4.5	Brown discoloration on entire root system with a shriveled, brown, and soft hypocotyl		
5.0	Dead plant		

**Table 2:** Dataset manipulation and evaluation procedure for assessing the DeepARRNet model and different class-balancing methods.

Dataset and Class-Balancing Technique Implemented	1st Seed (S_ia_)	2nd Seed (S_ib_)	3rd Seed (S_ic_)
S_1_—Without class balancing (original dataset)	Evaluate on S_1a_ (training with R_1a_ and test on T_1a_)	Evaluate on S_1b_ (training with R_1b_ and test on T_1b_)	Evaluate on S_1c_ (training with R_1c_ and test on T_1c_)
S_2_—Random oversampling	Evaluate on S_2a_ (training with R_2a_ and test on T_2a_)	Evaluate on S_2b_ (training with R_2b_ and test on T_2b_)	Evaluate on S_2c_ (training with R_2c_ and test on T_2c_)
S_3_—GAN-based image synthesis	Evaluate on S_3a_ (training with R_3a_ and test on T_3a_)	Evaluate on S_3b_ (training with R_3b_ and test on T_3b_)	Evaluate on S_3c_ (training with R_3c_ and test on T_3c_)
S_4_—Loss function with weighted ratio	Evaluate on S_4a_ (training with R_4a_ and test on T_4a_)	Evaluate on S_4b_ (training with R_4b_ and test on T_4b_)	Evaluate on S_4c_ (training with R_4c_ and test on T_4c_)

**Table 3:** Performance (Mean ± SD) during testing using DeepARRNet model trained with the original pea root images.

Class	Precision	Recall	F1-Score
Resistant	0.99 ± 0.02	0.92 ± 0.03	0.95 ± 0.03
Intermediate	0.80 ± 0.03	0.99 ± 0.03	0.88 ± 0.03
Susceptible	0.97 ± 0.05	0.06 ± 0.05	0.09 ± 0.05
Overall	0.93 ± 0.03	0.72 ± 0.03	0.83 ± 0.03

**Table 4:** Performance (Mean ± SD) during testing using DeepARRNet model trained with the original pea root and augmented data with random oversampling method.

Class	Precision	Recall	F1-Score
Resistant	0.99 ± 0.02	0.92 ± 0.03	0.96 ± 0.03
Intermediate	0.86 ± 0.04	0.98 ± 0.04	0.91 ± 0.04
Susceptible	0.91 ± 0.06	0.68 ± 0.06	0.78 ± 0.06
Overall	0.93 ± 0.03	0.85 ± 0.04	0.91 ± 0.04

**Table 5:** Performance (Mean ± SD) during testing using DeepARRNet model trained with the original pea root and GAN-augmented data.

Class	Precision	Recall	F1-Score
Resistant	0.99 ± 0.01	0.93± 0.01	0.96 ± 0.01
Intermediate	0.90 ± 0.05	0.99 ± 0.05	0.91 ± 0.05
Susceptible	0.91 ± 0.07	0.75 ± 0.04	0.81 ± 0.06
Overall	0.96 ± 0.03	0.87 ± 0.04	0.92 ± 0.033

**Table 6:** Performance (Mean ± SD) during testing using DeepARRNet model trained with the original pea root applying class weighing methods, INS and ISRNS.

Weight Ratio	Class	Precision	Recall	F1-Score
INS	Resistant	0.99 ± 0.01	0.93 ± 0.02	0.96 ± 0.02
	Intermediate	0.88 ± 0.05	0.98 ± 0.07	0.94 ± 0.06
	Susceptible	0.90 ± 0.08	0.64 ± 0.06	0.78 ± 0.07
	Overall	0.94 ± 0.04	0.85 ± 0.05	0.88 ± 0.05
ISRNS	Resistant	0.99 ± 0.03	0.93 ± 0.03	0.96 ± 0.03
	Intermediate	0.87 ± 0.06	0.98 ± 0.06	0.92 ± 0.06
	Susceptible	0.85 ± 0.07	0.60 ± 0.08	0.79 ± 0.07
	Overall	0.92 ± 0.05	0.83 ± 0.04	0.87 ± 0.05

## Data Availability

The data presented in this study are available on request from the corresponding author.

## Supplementary Materials

The following supporting information can be downloaded at: https://www.mdpi.com/article/10.3390/s22197237/s1, Table S1: Summary of GAN-generator model (layer-wise); Table S2: Summary of GAN-discriminator model (layer-wise); Table S3: Performance (Mean ± SD) during training with the original pea root images using DeepARRNet model; Table S4: Performance (Mean ± SD) during training with the original pea root images and random oversampling augmented data using DeepARRNet model; Table S5: Performance (Mean ± SD) during training with the original pea root and GAN-augmented data using DeepARRNet model; Table S6: Performance (Mean ± SD) during training with the original pea root applying class weighing methods, INS and ISRNS, using DeepARRNet model.

## Abbreviations

ARR—Aphanomyces root rot, CNN—convolutional neural network, GAN—Generative adversarial network, t-Conv—transposed convolutional, S_1_ to S_4_—datasets, T_1_ to T_4_—test datasets, R_1_ to R_4_—training datasets, INS—Inverse of number of samples, ISRNS—Inverse of square root of number of samples, and MBConv—mobile inverted bottleneck convolution.
