# Supplementary material for: Evaluation of Effective Class-Balancing Techniques for CNN-Based Assessment of Aphanomyces Root Rot Resistance in Pea (Pisum sativum L.)
Source: Sensors (Basel). 2022 Sep 24;22(19):7237. doi: 10.3390/s22197237 (PMC9572822; doi:10.3390/s22197237)
Supplement: Supplementary file 1 [file sensors-22-07237-s001.zip › sensors-1910861-supplementary.pdf]

Supplementary Materials

Table S1. Summary of GAN-generator model (layer-wise).

Layer Name	Parameter	Activations
Vector input		1 × 1 × 100
Fully connected		24 × 24 × 512
Transposed convolution	5 × 5 filters; 2 × 2 stride	28 × 28 × 256
Batch normalization	256 channels	28 × 28 × 256
ReLU	ReLU activation function	28 × 28 × 256
Transposed convolution	5 × 5 filters; 2 × 2 stride	56 × 56 × 128
Batch normalization	128 channels	56 × 56 × 128
ReLU	ReLU activation function	56 × 56 × 128
Transposed convolution	5 × 5 filters; 2 × 2 stride	112 × 112 × 64
Batch normalization	64 channels	112 × 112 × 64
ReLU	ReLU activation function	112 × 112 × 64
Transposed convolution	5 × 5 filters; 2 × 2 stride	224 × 224 × 3
tanh	tanh activation function	224 × 224 × 3

Table S2. Summary of GAN-discriminator model (layer-wise).

Layer Name	Parameter	Activations
Image input		224 × 224 × 3
Dropout	50% dropout	224 × 224 × 3
Convolution	5 × 5 filters; 2 × 2 stride	112 × 112 × 64
Leaky ReLU	0.20 scale	112 × 112 × 64
Convolution	5 × 5 filters; 2 × 2 stride	56 × 56 × 128
Batch normalization	128 channels	56 × 56 × 128
Leaky ReLU	0.20 scale	56 × 56 × 128
Convolution	5 × 5 filters; 2 × 2 stride	28 × 28 × 256
Batch normalization	256 channels	28 × 28 × 256
Leaky ReLU	0.20 scale	28 × 28 × 256
Convolution	5 × 5 filters; 2 × 2 stride	14 × 14 × 512
Batch normalization	512 channels	14 × 14 × 512
Leaky ReLU	0.20 scale	14 × 14 × 512
Convolution	5 × 5 filters; 2 × 2 stride	7 × 7 × 1024
Batch normalization	1024 channels	7 × 7 × 1024
Leaky ReLU	0.20 scale	7 × 7 × 1024
Convolution	5 × 5 filters; 2 × 2 stride	4 × 4 × 1024
Batch normalization	2048 channels	4 × 4 × 1024
Leaky ReLU	0.20 scale	4 × 4 × 1024
Convolution	4 × 4 filters; 1 × 1 stride	1 × 1 × 1

Table S3. Performance (Mean ± SD) during training with the original pea root images using DeepARRNet model.

Class	Precision	Recall	F1-score
**Resistant**	0.98 ± 0.03	0.94 ± 0.04	0.96 ± 0.04
**Intermediate**	0.85 ± 0.05	0.98 ± 0.02	0.92 ± 0.04
**Susceptible**	0.98 ± 0.07	0.32 ± 0.08	0.67 ± 0.07
**Overall**	**0.93 ± 0.05**	**0.78 ± 0.04**	**0.85 ± 0.04**

Table S4. Performance (Mean ± SD) during training with the original pea root images and random oversampling augmented data using DeepARRNet model.

Class	Precision	Recall	F1-score
**Resistant**	0.98 ± 0.04	0.94 ± 0.04	0.96 ± 0.04
**Intermediate**	0.89 ± 0.03	0.97 ± 0.05	0.93 ± 0.04
**Susceptible**	0.92 ± 0.07	0.74 ± 0.06	0.81 ± 0.06
**Overall**	**0.95 ± 0.05**	**0.88 ± 0.05**	**0.91 ± 0.05**

Table S5. Performance (Mean ± SD) during training with the original pea root and GAN-augmented data using DeepARRNet model.

Class	Precision	Recall	F1-score
**Resistant**	0.98 ± 0.03	0.93 ± 0.03	0.96 ± 0.03
**Intermediate**	0.91 ± 0.05	0.97 ± 0.07	0.94 ± 0.07
**Susceptible**	0.92 ± 0.07	0.79 ± 0.07	0.85 ± 0.07
**Overall**	**0.97 ± 0.05**	**0.89 ± 0.06**	**0.93 ± 0.06**

Table S6. Performance (Mean ± SD) during training with the original pea root applying class weighing methods, INS and ISRNS, using DeepARRNet model.

Weight ratio	Class	Precision	Recall	F1-score
**INS**	Resistant	0.98 ± 0.04	0.93 ± 0.05	0.96 ± 0.05
	Intermediate	0.90 ± 0.07	0.97 ± 0.08	0.93 ± 0.07
	Susceptible	0.91 ± 0.08	0.69 ± 0.08	0.70 ± 0.08
	**Overall**	**0.94 ± 0.07**	**0.90 ± 0.06**	**0.92 ± 0.07**
**ISRNS**	Resistant	0.98 ± 0.04	0.92 ± 0.07	0.95 ± 0.06
	Intermediate	0.88 ± 0.07	0.94 ± 0.08	0.92 ± 0.08
	Susceptible	0.90 ± 0.07	0.70 ± 0.08	0.80 ± 0.08
	**Overall**	**0.93 ± 0.05**	**0.86 ± 0.08**	**0.90 ± 0.07**
